# Plane wave imaging combined with eigenspace-based minimum variance beamforming using a ring array in ultrasound computed tomography

**DOI:** 10.1186/s12938-019-0629-2

**Published:** 2019-01-23

**Authors:** Xinming Jiang, Yang Xiao, Yuanyuan Wang, Jinhua Yu, Hairong Zheng

**Affiliations:** 1 Department of Electronic Engineering, Fudan University, Shanghai, China; 2 Shenzhen Institutes of Advanced Technology, Chinese Academy of Sciences, Shenzhen, China; 3 Key Laboratory of Medical Imaging Computing and Computer Assisted Intervention of Shanghai, Fudan University, Shanghai, China

**Keywords:** Ultrasound computed tomography, Echo image, Plane wave, Eigenspace-based minimum variance beamforming, Time-saving

## Abstract

**Background:**

Ultrasound computed tomography (USCT) is usually realized with a ring array. It can provide better imaging performance and more tissue information by emitting and receiving the ultrasound signal in different directions simultaneously. However, USCT imaging is usually applied with the synthetic aperture (SA) emission method, which leads to a long scanning time with a large number of elements on the ring array. The echo image can provide the structural information, and has a higher resolution than maps of other parameters in USCT. Hence, we proposed plane wave (PW) imaging for ring array to acquire the echo wave and reduce the scanning time considerably.

**Results:**

In this paper, an emitting and receiving process was proposed to realize plane wave imaging with a ring array. With the proposed scanning method, the number of emission events can be reduced greatly. A beamforming method based on the eigenspace-based minimum variance (ESBMV) was also combined with the scanning method. With ESBMV beamformer, the resolution and contrast ratio of reconstruction result can be maintained or even improved under a fewer-emissions condition. We validated the method using both computer simulations with Field II and phantom experiments with a ring array of 512 elements. The Verasonics^®^ system was used to transmit and receive the ultrasound signal in the phantom experiments.

**Conclusions:**

According to the results of the experiments, the imaging results will have a better contrast ratio with a higher emitting energy. Additionally, the scanning time with the proposed method can be only one-tenth of that with the SA emission method, while the echo imaging performance still remains at a similar level or even better.

## Introduction

Due to its real-time and non-ionizing properties and low cost, ultrasound imaging is widely used in the clinic. In conventional clinical ultrasound imaging, a linear or convex probe is usually used. However, there are several limitations. When imaging with linear or convex probes, ultrasound signal can be emitted and received in one direction at one time. Also, the ultrasound beam widens and attenuates greatly in far field, where there will be anisotropy in resolution and low image quality. Additionally, the scanning in traditional imaging based on a linear or convex probe is highly operator-dependent, which may easily introduce misdiagnosis due to scanning inappropriate planes. The ring array can naturally solve or produce some improvement in these problems because of its geometrical structure [1]. The ring array can receive not only the echo wave but also the scatter wave and transmission wave in different directions at the same time. Imaging with a ring array, we can get the structure information with the echo wave [1], as well as the maps of speed of sound and attenuation with the transmission wave [2–7]. Combining the above advantages, ultrasound computed tomography (USCT) has received increasing attention.

In USCT, the synthetic aperture (SA) emission method is applied to acquire both the transmission waves [3–5] and the echo waves [6, 7]. In the SA method, each element serves as a transmitter in a single emission event, while all elements act as receivers, and all elements emit in sequence. Obviously, in the SA emission method, the number of emission events is equal to that of elements on the ring array, which is usually a large number, such as 512 or more. Additionally, there may be severe multiple reflections inside the ring array after each emission event, while the energy will spread away soon in conventional B-mode imaging. Hence, there should be a time interval between two emission events, to ensure the ultrasound waves inside the ring array attenuates to an ignorable level. Therefore, the scanning time may be long enough to cause artefacts brought on by the breathing movements of patients. However, most research focused on the reconstruction algorithm for echo images and transmission images while few have focused on how to reduce the long scanning time in USCT, which may have a greater effect in clinical application. As mentioned above, the images reconstructed by the echo wave contain the significant structural information. In this paper, we mainly focus on accelerating the imaging process for echo imaging.

Obviously, the scanning time for each emitting event is restricted by the speed of sound and attenuation rate, which is hard to decrease. So we tried to reduce the number of emission events in each imaging process to increase the frame rate. To reduce the number of emission events, the plane wave (PW) emission method can be applied in the imaging process. The PW emission method was originally used with a linear array, accelerating the imaging speed in B-mode imaging. Instead of sweeping the imaging region line-by-line under the conventional scanning mode or emitting ultrasound waves element-by-element in the SA method, the plane wave transmitting method can scan the whole region with only one or several emission events. Here, the PW emission method is applied on the ring array instead of the SA emission method to reduce the scanning time of USCT.

Due to the lack of focusing in the process of pulse emission and the widening of the transmit beam, degradation of the image quality is still a problem for PW imaging [8]. To maintain the imaging result, in most PW beamforming methods, the plane wave will be emitted in different directions, also called steering angles [8–10]. However, the reconstruction area is not the same in different emission events in the proposed method, which means the plane wave compounding based on different steering angles are not appropriate for the proposed emission method. Some studies also use the harmonic component to improve the imaging result, which requires a ring array with a broad frequency response [11–13]. Minimum variance (MV) beamformer is an adaptive beamformer, and can be used in a single emission. MV beamformer can give a robust estimation and reduce the influence of sound speed errors [14]. Furthermore, MV beamformer can be applied with a smaller aperture size, and increase the imaging depth [15], which is ideal for ring arrays with a great number of elements and a large imaging area. In this study, we combine the emission method with the eigenspace-based minimum variance (ESBMV) beamforming method in [16–18], which was proposed based on the MV method and specifically yields higher contrast than the MV method.

In “Method” section, we describe our proposed method, including the emitting and receiving method, as well as ESBMV beamforming method. The settings of computer simulations and the phantom experiments are described in “Experiment” section. The results and discussions are included in “Results and discussion” section.

## Method

When emitting plane waves with a linear array, all elements are triggered simultaneously, while the time delay of each element will be designed individually when emitting with a ring array. In this section, the PW emitting and receiving methods based on ring arrays are introduced. The MV and ESBMV beamforming methods are then mentioned briefly.

### Plane wave transmitting method

Considering a ring array with $N$ elements in total, it is obvious that not all $N$ elements will be triggered in a single emission event to form a plane wave. Here, $N^{\prime}$ ($N^{\prime} < N$) elements are used in each emission event to produce the plane wave and the time delay $t_{delay} \left( i \right)$ of the $i th$ element of each transmitting aperture can be calculated as follows.

1 $$t_{delay} \left( i \right) = \left[ {r - r*\cos \left( {\left| {\frac{{N^{\prime} + 1}}{2} - i} \right|*\Delta \theta } \right)} \right]/c \quad i = 1,2, \ldots ,N^{\prime}$$

where $r$ is the radius of the ring array and $c$ is the speed of sound, which is considered to be a constant throughout the imaging area. $\Delta \theta$ is the central angle between two adjacent elements, as the elements of the ring array are uniformly distributed.

In each emission event, the plane wave can be formed in front of $N^{\prime}$ elements, and the area in front of the $N^{\prime}$ elements can be scanned and reconstructed. The transmitting aperture can be shifted around the target to yield a whole view. For a shift of $\Delta N$ elements between two adjacent emission events, the total number of plane wave emission events $M$ will be calculated as follows:

2 $$M = floor\left( {\frac{N}{\Delta N}} \right) + 1$$

where $floor\left( {\frac{N}{\Delta N}} \right)$ rounds the $\frac{N}{\Delta N}$ to the nearest integers less than or equal to $\frac{N}{\Delta N}$. Obviously, $M$ is much smaller than $N$, which means that the scanning time with the plane wave method can be $\frac{M}{N}$ of the time with the SA method.

### Receiving method

In the $j th$ emission event, $N^{\prime}$ elements are triggered by order according to the time delay mentioned in Eq. (1). The element in the centre of the transmitting aperture is triggered first ($t = 0$), and the plane wave is formed completely after the time $t = \mathop {\hbox{max} }\nolimits_{i} \left\{ {t_{delay} \left( i \right)} \right\}$, which means that the receiving signal before time $t = \mathop {\hbox{max} }\nolimits_{i} \left\{ {t_{delay} \left( i \right)} \right\}$ cannot be used and may cause a dead zone. However, the dead zone is relatively small and can be avoided in application by placing the target in the centre of the ring array.

For a certain position $\varvec{p}$ in front of the $j th$ transmitting aperture, the plane wave will reach $\varvec{p}$ when the time is

3 $$t_{j} = \frac{{r + \varvec{p} \cdot \varvec{d}_{j} }}{c}$$

**Fig. 1:**
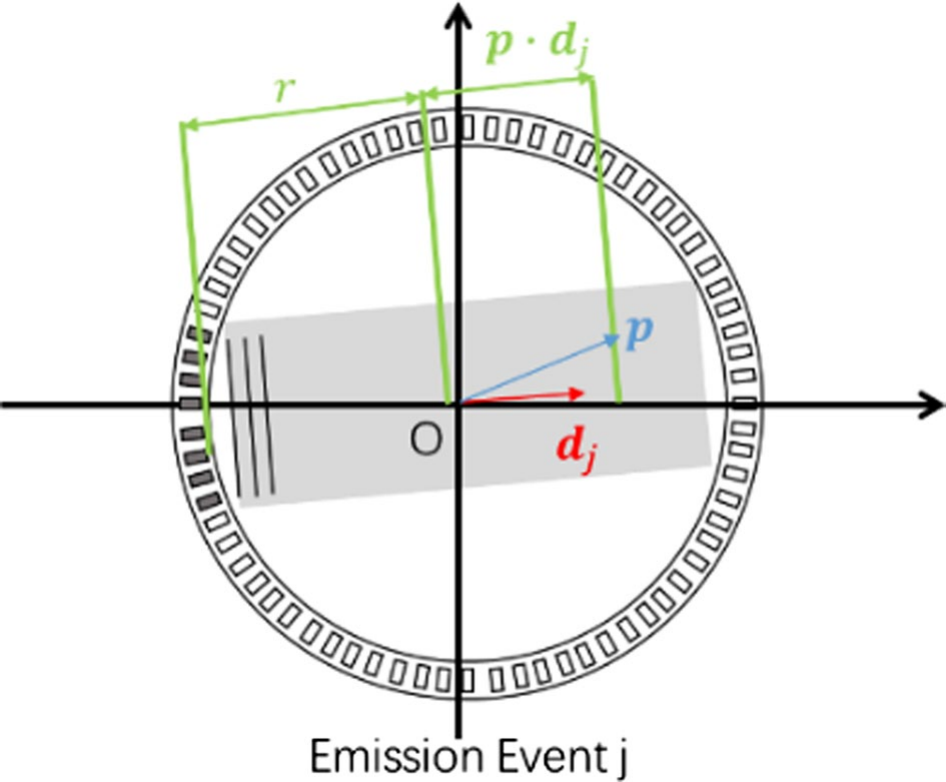
The position $\varvec{p}$ and direction $\varvec{d}_{j}$ in Eqs. 3 and 4

The echo wave from the position $\varvec{p}$ can be received by the $i th$ element at time

4 $$t_{i,j} = \frac{{r + \varvec{p} \cdot \varvec{d}_{j} }}{c} + \frac{{\left| {\varvec{p} - \varvec{e}_{i} } \right|}}{c}$$

where $\varvec{d}_{j}$ is a unit vector that represents the spreading direction of the plane wave in the $j th$ emission event, as shown in Fig. 1, and $\varvec{e}_{i}$ is the position of the $i th$ element on the transmitting aperture. The origin of the two vectors $\varvec{d}_{j}$ and $\varvec{e}_{i}$ is the same as the geometry centre of the ring array.

**Fig. 2:**
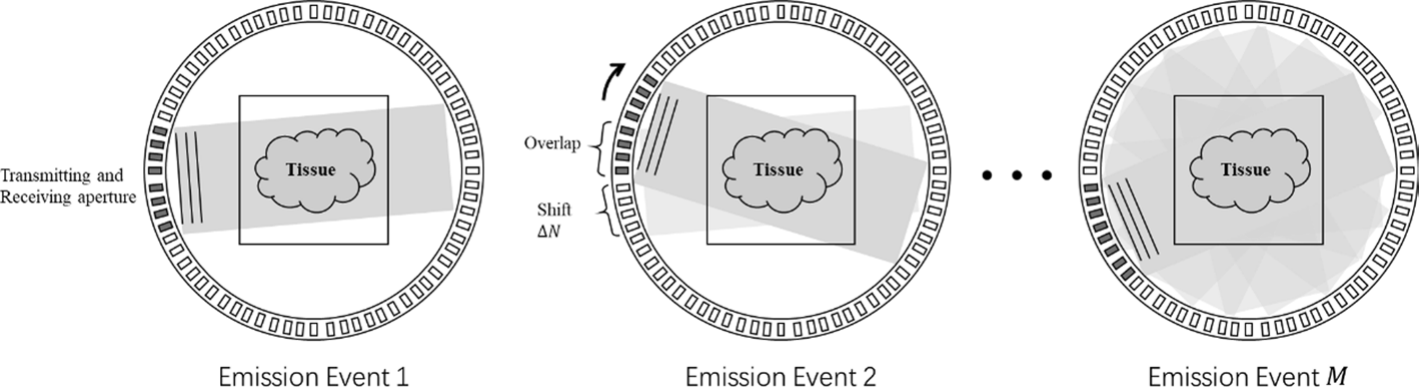
The $M$ emission processes with a ring array

Thus, the area in front of the $j th$ transmitting aperture can be reconstructed. The whole result can also be calculated after all $M$ emission events are finished. The total $M$ emission processes are shown in Fig. 2.

### Eigenspace-based minimum variance beamforming for a ring array

Minimum variance is the most commonly used adaptive beamforming method. Instead of being added up easily as in the delay-and-sum (DAS) method, the signals from different elements have different weights applied before being added. The weight is calculated according to the received signal. Here, the MV beamformer is briefly introduced according to [14–19]. In a certain emission event, $N^{\prime}$ elements of the ring array are used as both transmitters and receivers, so the signal received from each element can be arranged as a vector $\varvec{X}$ after the time delay at position $\varvec{p}$.

5 $$\varvec{X}\left( \varvec{p} \right) = \left[ {x_{1} ,x_{2} , \ldots ,x_{{N^{\prime}}} } \right]^{T}$$

Then, the final output for a certain position reconstructed by this emission is

6 $$z\left( \varvec{p} \right) = \varvec{W}^{H} \cdot \varvec{X} = \mathop \sum \limits_{i = 1}^{{N^{\prime}}} w_{i}^{*} \cdot x_{i}$$

where $x_{i}$ is the sample received at the $i th$ element and $w_{i}$ is the adaptive weight on the $i th$ element in this emission event. The $N^{\prime}$ different weights can also be arranged as a vector.

7 $$\varvec{W} = \left[ {w_{1} ,w_{2} , \ldots ,w_{{N^{\prime}}} } \right]^{T}$$

$\cdot^{H}$ is the conjugate transpose, and $\cdot^{ *}$ represents the complex conjugate.

In the MV method, the optimization problem can be expressed as

8 $$\begin{aligned} {\text{min }}E\left[ {z^{2} } \right] =\, & \hbox{min} \varvec{W}^{H} \varvec{RW} \\ {\text{s}}.{\text{t}}. \varvec{W}^{H} \cdot \varvec{d} =\, & 1 \\ \end{aligned}$$

where $\varvec{d}$ is the steering vector and will become $\varvec{d} = \left[ {1,1, \ldots ,1} \right]^{T}$ after the time delay. The covariance matrix $\varvec{R}$ is defined as $\varvec{R} = E\left[ {\varvec{X} \cdot \varvec{X}^{H} } \right]$. The solution to the MV problem is calculated by

9 $$\varvec{W}_{MV} = \frac{{\varvec{R}^{ - 1} \varvec{d}}}{{\varvec{d}^{H} \varvec{R}^{ - 1} \varvec{d}}}$$

The covariance matrix $R$ is not given directly. In practice, $R$ is estimated with the receiving signal as in Eq. (10).

10 $$\hat{\varvec{R}} = \frac{1}{{N^{\prime} - L + 1}}\mathop \sum \limits_{q = 1}^{{N^{\prime} - L + 1}} {\text{x}}_{q} {\text{x}}_{q}^{H}$$

where $L$ is the length of the subarray and ${\text{x}}_{q} = \left[ {x_{q} ,x_{q + 1} , \ldots ,x_{q + L - 1} } \right]^{T}$ is the signal vector on the $q th$ subarray. To make the result more robust, diagonal loading is also applied in estimating $\hat{R}$, in the form of $\varepsilon \varvec{I}$.

11 $$\varepsilon = \frac{1}{{100*N^{\prime}}}*tr\left\{ {\hat{\varvec{R}}} \right\}$$

where, $tr\left\{ \cdot \right\}$ is the trace of matrix $\hat{\varvec{R}}$. Then, the estimated covariance matrix $\hat{\varvec{R}}$ is applied to calculate the adaptive weight $\varvec{W}_{MV}$.

To further improve the reconstruction result, the covariance matrix $\varvec{R}$ can be eigen-decomposed into two orthogonal subspaces, called the signal subspace $\varvec{R}_{s}$ and the noise subspace $\varvec{R}_{n}$, in the ESBMV beamformer [16].

12 $$\varvec{R} = \varvec{U\varLambda U}^{H} = \mathop \sum \limits_{i = 1}^{L} \lambda_{i} \varvec{v}_{i} \varvec{v}_{i}^{H} = \mathop \sum \limits_{i = 1}^{{N_{sig} }} \lambda_{i} \varvec{v}_{i} \varvec{v}_{i}^{H} + \mathop \sum \limits_{{i = N_{sig} + 1}}^{L} \lambda_{i} \varvec{v}_{i} \varvec{v}_{i}^{H} = \varvec{U}_{s}\varvec{\varLambda}_{s} \varvec{U}_{s}^{H} + \varvec{U}_{p}\varvec{\varLambda}_{p} \varvec{U}_{p}^{H} = \varvec{R}_{s} + \varvec{R}_{n}$$

where $\varvec{\varLambda}= {\text{diag}}\left[ {\lambda_{1} ,\lambda_{2} , \ldots ,\lambda_{L} } \right]$ are the eigenvalues arranged in descending order and $\varvec{U} = \left[ {\varvec{v}_{1} ,\varvec{v}_{2} , \ldots ,\varvec{v}_{L} } \right]$ is composed of the $L$ orthonormal eigenvectors $\varvec{v}_{i}$ corresponding to the eigenvalues $\lambda_{i}$, while the signal subspace $\varvec{U}_{s}$ is composed of $N_{sig}$ orthonormal eigenvectors $\varvec{v}_{i}$ corresponding to the $N_{sig}$ largest eigenvalues $\lambda_{i}$. Then, the adaptive weights in the ESBMV beamformer are calculated as

13 $$\varvec{W}_{ESBMV} = \varvec{U}_{s} \varvec{U}_{s}^{H} \varvec{W}_{MV}$$

The final imaging result can be reconstructed by adding the $M$ results of all emission events.

## Experiment

To validate the proposed method, we performed both computer simulations and phantom experiments. Computer simulations and all image reconstructions were performed in MATLAB R2017b (MathWorks, Inc., Natick, MA, United States).

### Computer simulation

The echo signal of both the SA and PW emitting methods is simulated using Field II [20, 21]. The simulated ring array has 540 elements ($N$), with a radius ($r$) of 99 mm. The elements are uniformly distributed over 360° such that the central angle between two adjacent elements ($\Delta \theta$) is approximately 0.67°. The ring array has a centre frequency of 1 MHz and a sample rate of 10 MHz. The excitation signal is composed of a sine signal (2 cycles).

**Fig. 3:**
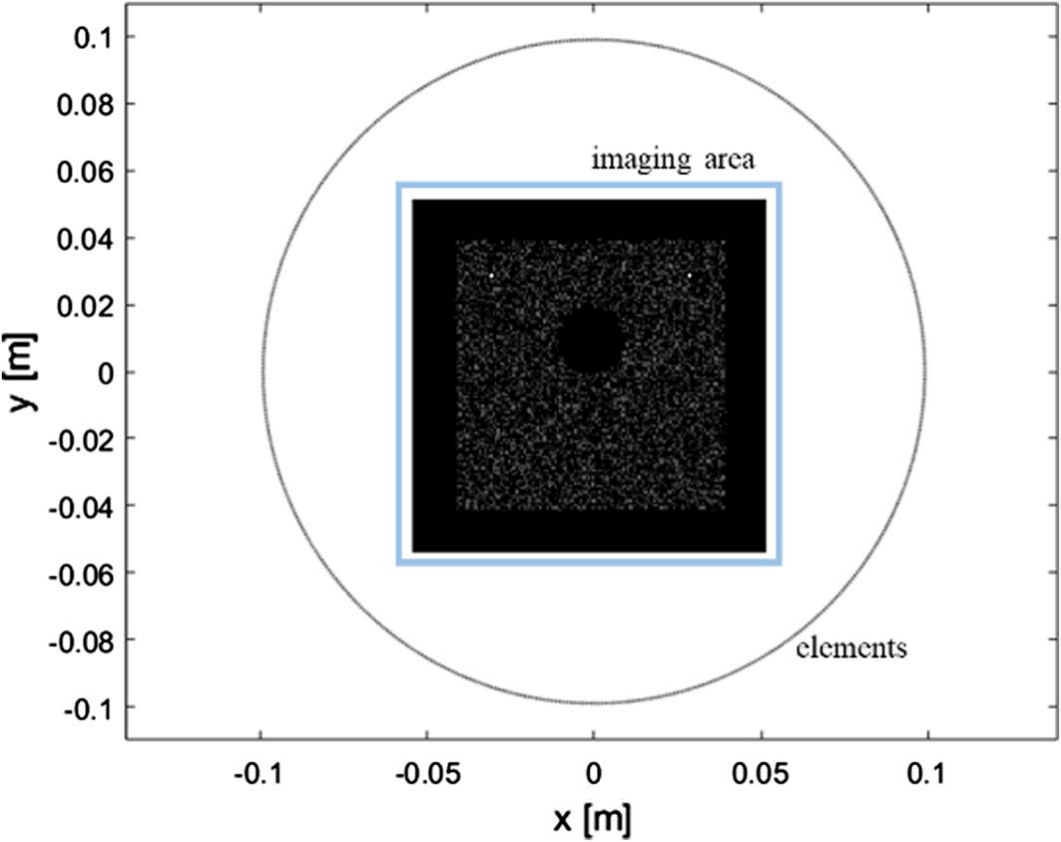
Mapping of elements and the schematic of the computer simulation phantom

The phantom is shown in Fig. 3. A square with a length of 80 mm is positioned in the centre of the ring array. Ten thousand background scatterers are randomly positioned inside the square, except for inside the circle. The circle has a radius of 10 mm and a distance of 10 mm away from the centre of the square, representing a cyst. Two point targets are positioned at (− 0.03 m, 0.03 m) and (0.03 m, 0.03 m), with amplitudes of 10 times the background scatterers inside the square. Both the SA emission method and the plane wave emission method were realized with the same phantom.

In the SA emission method, Qu et al. in [1] reconstructed an image with half of all elements as the receivers in each emission event. Since Field II cannot simulate a concave array with a central angle equal to or near 180°, we only used 135 elements $\left( {\frac{135}{540} *360^\circ = 90^\circ } \right)$ as the receivers in the simulation. We also simulated the SA emission method with 67 elements $\left( {\frac{67}{540} *360^\circ \approx 45^\circ } \right)$ for comparison with the aperture size of 64 elements using the proposed method.

In the plane wave simulation, the transmission focus $f_{tx}$ is set at a distance much larger than the radius of the concave array $r_{concave}$ $\left( {f_{tx} \gg r_{concave} = 99\;{\text{mm}}} \right)$ so that the transmitted wave can be approximately considered as a plane wave. The receiving focus $f_{rx}$ is set the same as $r_{concave}$, as no additional time delay is added to the receiving signal. Usually, the size of the transmit aperture is a power of 2. Here, we tried transmit apertures with 32 and 64 elements, respectively, while maintain $\Delta N$ as 8.

To qualify the performance of SA and plane wave emission on both point targets and the cyst, the full width at half maximum (FWHM) and the contrast ratio (CR) were measured. The FWHM represents the resolution of the image result. For consistency, we measured the width of the point target at (− 0.03 m, 0.03 m) along the y-axis as the FWHM. The CR was measured as the intensity ratio of the areas inside and outside the cyst as

14 $$CR = 20log_{10} \left( {\frac{{\mathop \smallint \nolimits_{{\varvec{\varOmega}_{in} }}^{ } I\left( \varvec{p} \right) \cdot d\varvec{p}}}{{\mathop \smallint \nolimits_{{\varvec{\varOmega}_{out} }}^{ } I\left( \varvec{p} \right) \cdot d\varvec{p}}}} \right)$$

where $I\left( \varvec{p} \right)$ is the amplitude at position $\varvec{p}$, while $\varvec{\varOmega}_{in}$ and $\varvec{\varOmega}_{out}$ are the areas inside and outside the cyst respectively.

### Real phantom experiment

**Fig. 4:**
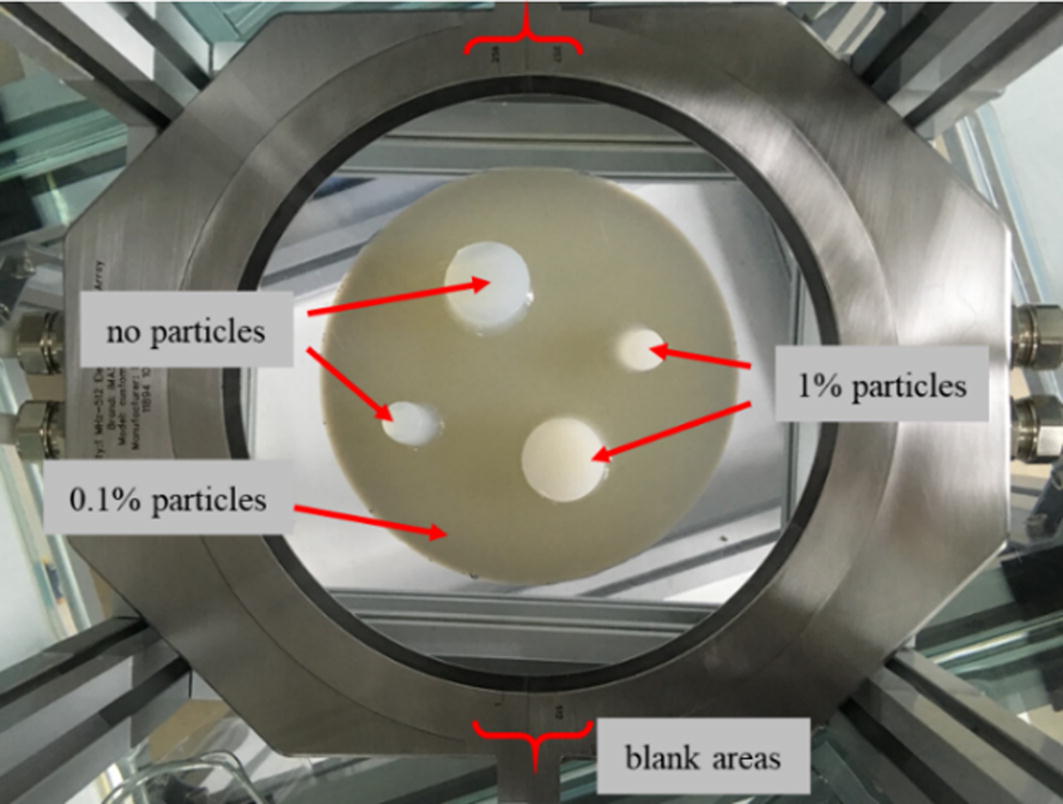
The ring array and model used in the experiment, placed in a glass water tank

The real model experiment was carried out on the Verasonics^®^ system. The ring array used is composed of two semicircle probes. Each probe has 256 elements uniformly distributed over 170°, with a 5° margin on both ends of each semicircle. Therefore, there is also 0.67° between two elements, the same as the settings in the computer simulation, except for the total of 20° blank areas in the whole ring array. The ring array and the phantom are shown in Fig. 4.

The ring array is driven by two Verasonics^®^ Vantage 256 systems simultaneously. In the real phantom experiment, the excitation voltage is set at 40 V and the sampling rate at 20 MHz. The centre frequency is 1 MHz, and the sampling depth is 5120 points in each channel, corresponding to a distance of approximately 0.39 m at 1540 m/s, which is approximately 4 times the radius of the ring array, ensuring that almost all of the reflected signal is received.

The model is composed of some right circular cylinders made of 1.5% agarose. As agarose does not greatly reflect the ultrasound wave, particles are added into the cylinders as scatterers at different concentrations. The particle is a kind of cross-linked polymethylmethacrylate spherical particle. It has good heat resistance and solvent resistance, and can maintain its characteristic in the hot water and in the phantom for some time. The particle has an average diameter of about 40 μm, which is similar to the size of cells in human body. The final phantom is also shown in Fig. 4. The largest cylinder consists of approximately 0.1% particles so that it can exhibit a weak reflection under the ultrasound probe. Two larger and two smaller cylinders are also positioned symmetrically inside the largest cylinder as shown in Fig. 4. No particles are added into one of the larger cylinders and one of the smaller cylinders so that these two cylinders will reflect no ultrasound signal and can be considered as cysts in the human body. The other two cylinders are composed of 1% particles and will have a stronger reflection than the surrounding materials. All cylinders are vertically positioned so that the imaging result will be 5 circles with different intensities.

In the real phantom experiment, the emission strategy is modified, as the elements of the ring array are not uniformly spaced. As mentioned in above, 256 elements are uniformly distributed over 170° of either semicircle probe. For the SA method, the transmitting process is not changed, except that the number of emission events is 512 instead of 540. With the plane wave method, 32 or 64 elements and also 40 elements, according to the simulation results, are used as transmitters and receivers in each emission event. Considering that it is hard to form a plane wave with blank areas in the transmitting aperture, the aperture is shifted only to where elements are distributed. For example, $$N$$, $$N^{\prime}$$ and $$\Delta N$$ are 512, 64 and 8, respectively. In the first event, element 1 to 64 will be triggered, followed by element 9 to 72, and so on. As there is a gap between element 256 and 257, only element 193 to 256 or element 257 to 319 will be triggered in their emission events, respectively. The sequence ends with element 449 to 512. Therefore, the number of emission events on either semicircle probe is $$\frac{{{\raise0.7ex\hbox{$N$} \!\mathord{\left/ {\vphantom {N 2}}\right.\kern-0pt} \!\lower0.7ex\hbox{$2$}} - N^{\prime}}}{\Delta N} + 1$$, and the total number of emission events will be

15 $$M = 2 *\left( {\frac{{{\raise0.7ex\hbox{$N$} \!\mathord{\left/ {\vphantom {N 2}}\right.\kern-0pt} \!\lower0.7ex\hbox{$2$}} - N^{\prime}}}{\Delta N} + 1} \right)$$

Provided that $N$, $N^{\prime}$ and $\Delta N$ are 512, 64 and 8, respectively, $M$ will be 50, and this value will be 58 with 32 elements as the aperture. Similarly, with 40 elements as the aperture, $M$ will also be 50 when $\Delta N$ is set at 9.

### Summary of experimental processes

Here, we summarize the processes of the computer simulation/phantom experiments. The parameters are shown, respectively in Table 1.

**Table 1:** Parameters in simulations and experiments

Parameter	Computer simulation	Phantom experiment	Parameter	Computer simulation	Phantom experiment
$N$	540	512	$f_{s}$	10 MHz	20 MHz
pitch	1.15 mm		Excitation signal	Sine (2 cycles)	Square (2 cycles)
kerf	0.2 mm		$N^{\prime}$	32, 64,…	
$R$	99 mm		$L$	$N^{\prime}/4$	
$$\Delta \theta$$	0.67°		Diagonal loading	$${\raise0.7ex\hbox{$1$} \!\mathord{\left/ {\vphantom {1 {100 *L}}}\right.\kern-0pt} \!\lower0.7ex\hbox{${100 *L}$}}$$	
$M$	68	58/50	Eigenvalue (signal)	$\lambda_{i} > 0.05*\lambda_{max}$	
$f_{c}$	1 MHz		Eigenvalue (noise)	$\lambda_{i} < 0.05*\lambda_{max}$

1. Phantom settings as in “Computer simulation” section/Making a model with agarose.
2. Transducer settings/Preparing the ring array and Verasonics^®^ system.
3. Calculating the time delay on each element.
4. Emitting and receiving ultrasound signals.
5. Reconstructing the image area according to “Method” section.

## Results and discussion

### Computer simulation result

**Fig. 5:**
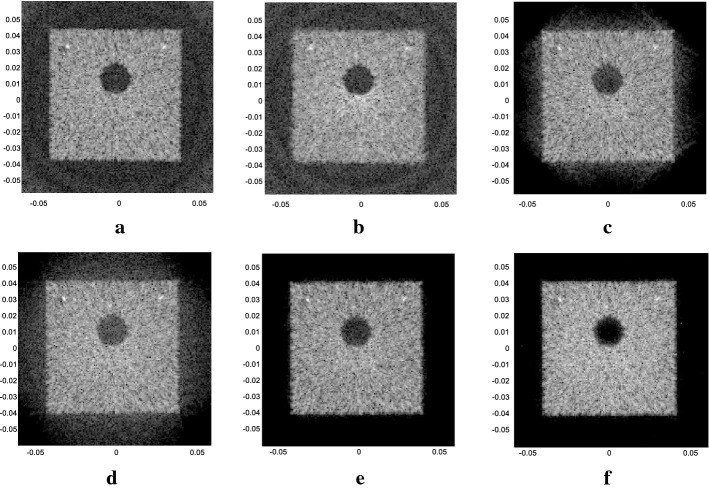
Results of different methods in the computer simulation. **a** The SA method with 67 elements as receivers. **b** The SA method with 135 elements as receivers. **c** The plane wave-DAS method with 32 elements. **d** The plane wave-DAS method with 64 elements. **e** The plane wave-ESBMV method with 32 elements. **f** The plane wave-ESBMV method with 64 elements

The computer simulation results are shown as Fig. 5. Figure 5a, b are the results for SA emission, with 67 and 135 elements as the receiving aperture. Figure 5c–f are the results for plane wave emission, with 32 and 64 elements as the aperture combining with DAS and ESBMV beamformer, respectively. As $\Delta N$ is 8, the number of emission events in each plane wave simulation (Fig. 5c–f) was 68 according to Eq. (2), while it was 540 for Fig. 5a, b. All simulation results displayed are over a dynamic range of 60 dB.

**Fig. 6:**
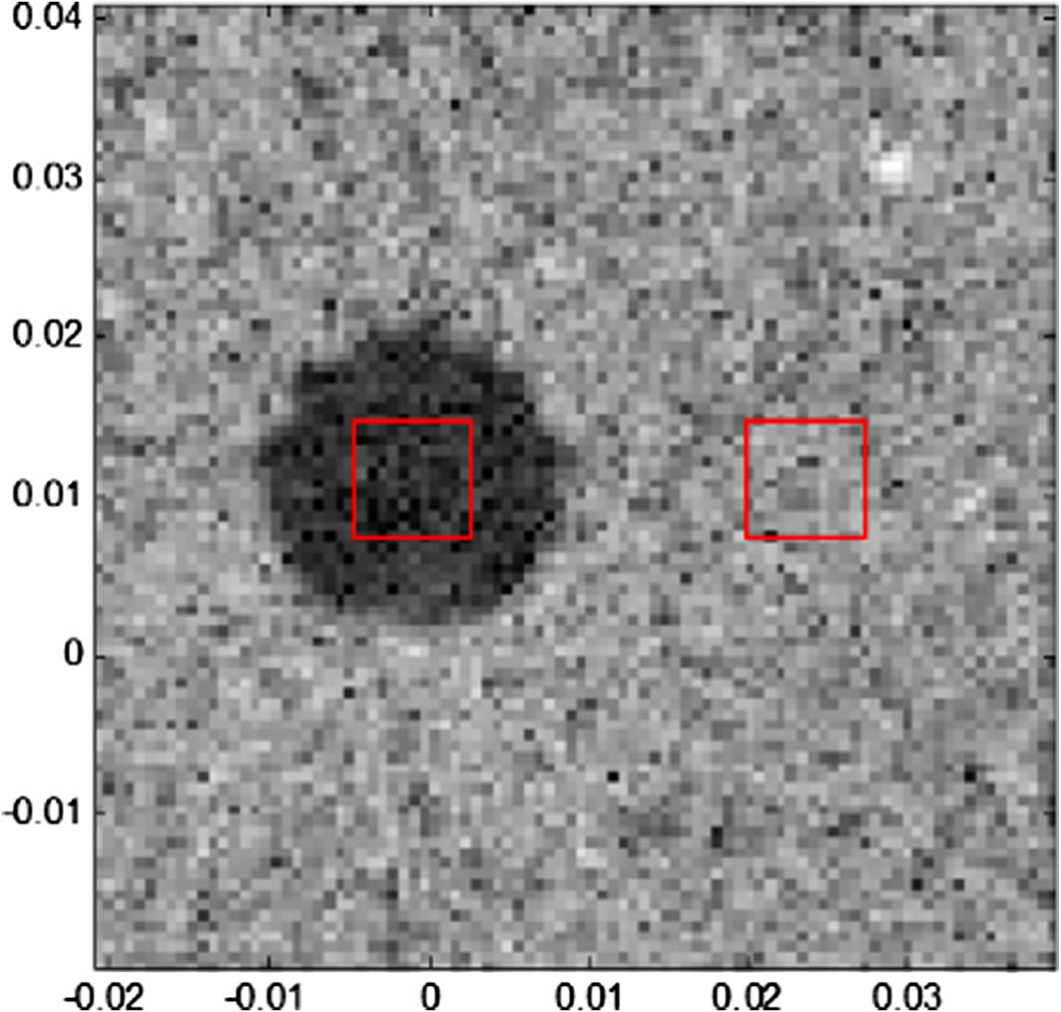
The areas inside and outside the cyst used in the calculation of CR

According to Fig. 5, the SA emission method will have some background artefacts even outside the square phantom, where there is no scatterer positioned and should be totally anechoic. The plane wave emitting method can certainly reduce the number of emission events and produce a lower level of background artefacts. Additionally, with the help of the ESBMV beamformer, the image quality can be improved greatly. The FWHM and CR are measured, and the areas $\varvec{\varOmega}_{in}$ and $\varvec{\varOmega}_{out}$ mentioned in (14) are shown in Fig. 6. The performances of different emission methods and parameters are shown in Table 2.

**Table 2:** Performance of both transmitting method with different parameters

Method	FWHM (mm)	CR (dB)
SA with 67 elements (Fig. 5a)	2.21	− 53.48
SA with 135 elements (Fig. 5b)	2.10	− 51.03
PW-DAS with 32 elements (Fig. 5c)	1.67	− 49.05
PW-DAS with 64 elements (Fig. 5d)	2.13	− 39.34
PW-ESBMV with 32 elements (Fig. 5e)	1.57	− 54.39
PW-ESBMV with 64 elements (Fig. 5f)	2.10	− 101.58

### Affects from aperture size and overlap

**Fig. 7:**
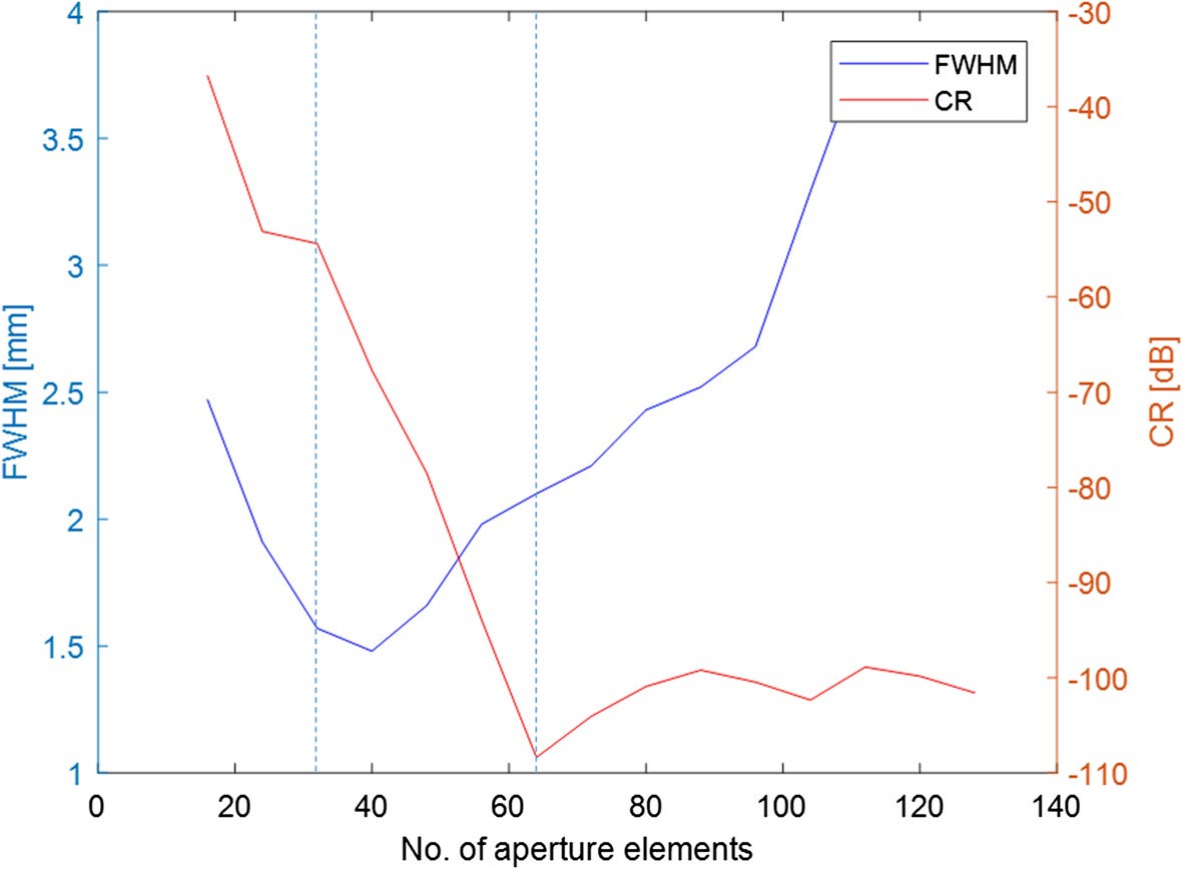
FWHM and CR of images with different aperture sizes obtained with the proposed method

Furthermore, in the plane wave emission method, the length of the aperture may have some impact on the performance for both point targets and the cyst according to the results in Fig. 5. To evaluate the performance with different aperture sizes, we also simulated the plane wave emitting method using from 16 elements to 128 elements, while maintain $\Delta N$ and $M$ as 8 and 68, respectively. The FWHM and CR of different aperture sizes can be seen in Fig. 7. The two vertical lines represent the results with 32 and 64 elements, respectively.

According to Fig. 7, using a small aperture less than 80 elements, there will be a better resolution (from FWHM) on the point target, while there will be a better contrast ratio (from CR) on the cyst target using a large aperture greater than about 40 elements. Here, the aperture size between 32 and 64 elements can be considered a suitable aperture size.

**Fig. 8:**
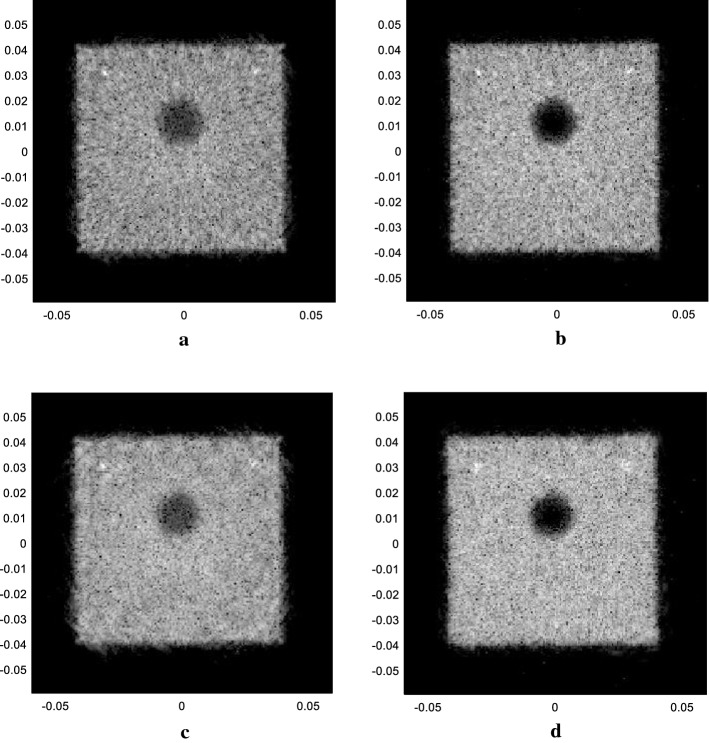
Results of the plane wave method in the computer simulation with half and one-fourth of the emission events. **a**, **c** The plane wave- ESBMV method with 32 elements. **b**, **d** The plane wave- ESBMV method with 64 elements. In **a**, **b**, $\Delta N$ is 16, while the value is 32 in **c**, **d**

We also simulated the plane wave transmitting method with fewer emission events. Here, $\Delta N$ is increased to 16 and 32 to reduce the value of $M$, which means that the overlap areas will be fewer or even none. Therefore, the scanning time will be half or one-fourth of the above. The results are shown in Fig. 8.

With fewer emission events, as we can see in Fig. 8, there will be less or even no overlap areas between different emission events. The image quality, especially that of the outer side of the images with fewer overlap areas, will decrease. This is the trade-off between the number of emission events controlled by $\Delta N$ and the image quality, which means that we should chose a medium aperture length combined with a relevant $\Delta N$ to obtain an ideal result.

### Real phantom experiment result

**Fig. 9:**
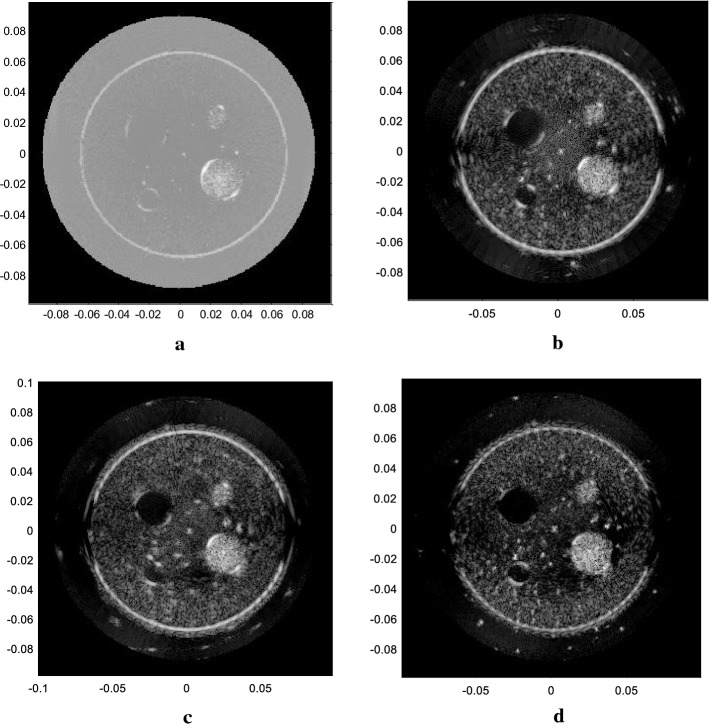
Results of different methods in the real model experiment. **a** The SA method with 67 elements as receivers. **b** The plane wave-ESBMV method with 32 elements. **c** The plane wave-ESBMV method with 40 elements. **d** The plane wave-ESBMV method with 64 elements

The results of the real phantom for the SA method and the plane wave method are displayed in Fig. 9. All the four figures are in a dynamic range of 50 *dB*. Figure 9a is the result for SA emission method, with 67 elements as the receiving aperture and 512 emission events. Figure 9b–d are the result for plane wave emission method, using 32, 40, 64 elements as the aperture combined with $\Delta N$ as 8, 9 and 8, respectively, the same as those mentioned in “Real phantom experiment” section.

Obviously, with only one element emitting in the SA method, the energy of the ultrasound pulse is limited. Some details may not be seen as shown in Fig. 9a, and the contrast between the background area and the anechoic area is relatively low. The two anechoic areas are even hard to distinguish in Fig. 9a. With more elements emitting in the plane wave emitting method, scatterers with weak reflection can be seen clearly. Additionally, with the ESBMV beamformer, the contrast ratio between the cyst and the surrounding areas is improved greatly. Similar to the simulation result, imaging with an appropriate aperture can bring improvement of both the point target and the cyst area.

**Fig. 10:**
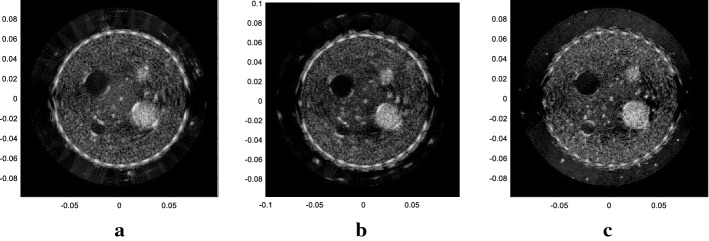
Results of different methods in the real model experiment with fewer emission events. **a** The plane wave-ESBMV method with 32 elements. **b** The plane wave-ESBMV method with 40 elements. **c** The plane wave-ESBMV method with 64 elements

Here, we also tried a larger $\Delta N$ with twice the original setting to produce a further reduction of the scanning time. $M$ reached to 26 (with an aperture size of 64 elements and 40 elements) and 30 (with an aperture size of 32 elements) here, and the results are shown in Fig. 10 with 50 dB dynamic range.

**Fig. 11:**
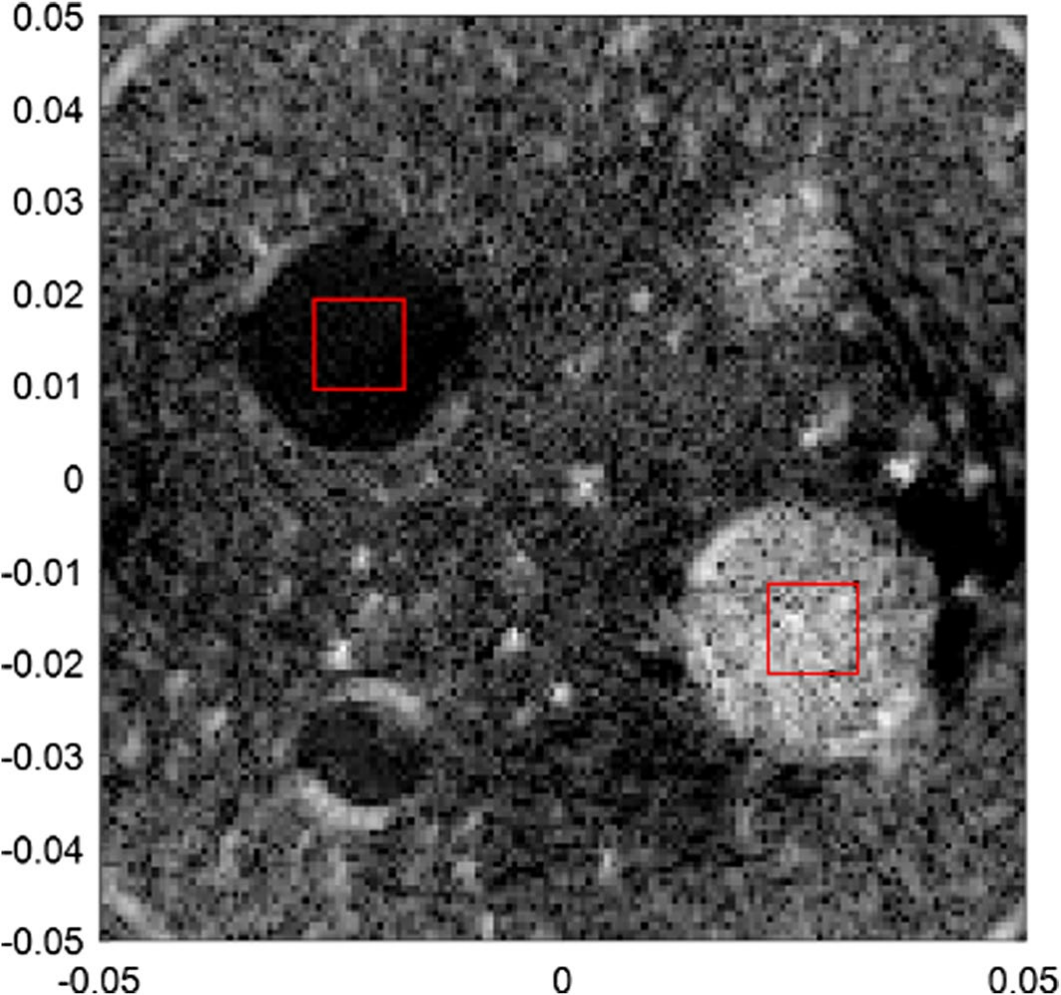
The areas with high energy and low energy used in the calculation of CR

In Fig. 10, with fewer emissions and overlap areas, there will be degradation of the performance, especially in the areas away from the image centre. We also measured the CR in the phantom experiment results (Figs. 9b, d, 10a, c, all with $\Delta N$ as 8), between the areas $\varvec{\varOmega}_{low energy}$ with $\varvec{\varOmega}_{high energy}$, as shown in Fig. 11.

Here, we didn’t measure the FWHM as we don’t know the real situation (such as shape or size) of the point targets, which were caused by air bubbles or cracks of the model. The evaluation is shown as in Table 3. The results show the slight differences in imaging result caused by the aperture size and the number of emission events, which also meets the results mentioned in “Affects from aperture size and overlap” section.

**Table 3:** Contrast ratio of the proposed method with different parameters

Method	CR (dB)
PW with 32 elements (Fig. 8b)	− 14.76
PW with 64 elements (Fig. 8d)	− 17.58
Fewer PW emitting with 32 elements (Fig. 9a)	− 14.74
Fewer PW emitting with 64 elements (Fig. 9c)	− 17.20

Finally, since the phantom used in the experiment is made only for temporary use, there are plenty of air bubbles and cracks inside the model, which may be greatly reflected the ultrasound. The air bubbles and small pieces dropping from the phantom may also act as particles randomly positioned outside the model. Additionally, the dark areas on the left and right sides of the image are brought about by the ‘blank’ area (no elements) on the ring array, where there is also a degradation in image performance. This can be reduced or eliminated when using a ring array with fewer or no blank areas. However, the reconstructions of the areas of strong and weak reflection still showed great improvement. With the proposed plane wave emission method, the scanning time can be decreased to only one-tenth of that of the original SA method, and the reconstruction performance will remain similar or be even better.

## Conclusion

The low frame rate with the SA emission method greatly restricts the use, and the imaging result of the ring array. Therefore, in this paper, we tried to use the plane wave emission method on the ring array, which was originally used with a linear probe, to accelerate the scanning time for echo image. To maintain the image quality, we combined the plane wave emission method with the ESBMV beamformer. We validated our method via computer simulation based on Field II and through real phantom experiments based on a Verasonics^®^ system. According to the results, the emission events can be up to one-tenth of those in the original SA emission method, with a similar or even better image performance.

## Abbreviations

CR: contrast ratio
DAS: delay-and-sum
ESBMV: eigenspace-based minimum variance
FWHM: full width at half maximum
MV: minimum variance
PW: plane wave
USCT: ultrasound computed tomography
SA: synthetic aperture
